# Can titanium miniplates provide superior fixation compared to reconstruction plates in mandibular repair with iliac crest flaps? A retrospective study

**DOI:** 10.3389/fbioe.2025.1688458

**Published:** 2025-12-02

**Authors:** Wen-qing Lin, Da Liu, Renbin Zhou, Hao Lin, Bang Zeng, Jun Jia, Yifang Zhao, Bing Liu, Tianfu Wu

**Affiliations:** 1 Department of Oral and Maxillofacial Surgery, Stomatological Hospital of Xiamen Medical College, Xiamen, China; 2 Xia-men Key Laboratory of Stomatological Disease Diagnosis and Treatment, Xiamen, Fujian, China; 3 State Key Laboratory of Oral & Maxillofacial Reconstruction and Regeneration, Key Laboratory of Oral Biomedicine Ministry of Education, Hubei Key Laboratory of Stomatology, School & Hospital of Stomatology, Wuhan University, Wuhan, China; 4 Department of Oral and Maxillofacial Head Neck Surgery, School & Hospital of Stomatology, Wuhan University, Wuhan, China

**Keywords:** titanium miniplate, reconstruction plate, mandibular reconstruction, vascularized iliac crest flap, complication, fixation method, mini plate, quality of life

## Abstract

This study retrospectively analyzed 112 patients who underwent mandibular reconstruction with vascularized iliac crest flaps using different fixation strategies in routine clinical practice. Under comparable defect lengths and heights, reconstruction using mini plates was associated with the shortest operative time (p < 0.001), while postoperative drainage duration showed no significant difference among groups (p = 0.958). The number of iliac bone segments varied significantly among fixation methods: reconstruction plates (alone or combined with mini plates) were predominantly applied in multi-segmental reconstructions, whereas mini plates were mainly used for single-segmental repairs. Postoperative quality of life was evaluated using the University of Washington Quality of Life Questionnaire (UW-QoL). Although the fixation methods were selected according to defect complexity and surgical requirements, all approaches achieved satisfactory outcomes, and no significant difference in overall quality-of-life scores was observed among the three groups (p = 0.354). However, a significant difference was observed in the “Swallowing” domain, with patients in the mini plate group reporting better swallowing function (p = 0.009).

## 1 Introduction

The mandible is a critical bony structure of the maxillofacial region, responsible for functions such as mastication, speech, and facial aesthetics. Segmental defects resulting from tumors, trauma, infections, or congenital malformations not only impair patients’ functional and aesthetic outcomes but may also lead to psychological distress (Pickrell and Hollier, 2017). Vascularized iliac crest flap transplantation, owing to its abundant bone volume, reliable blood supply, and excellent shaping capability, has emerged as one of the most preferred methods for mandibular defect reconstruction (Yang et al., 2023). This technique, utilizing iliac crest flaps pedicled on the deep circumflex iliac vessels, effectively restores mandibular continuity and function while enabling the simultaneous repair of soft tissue defects with skin islands or internal oblique muscles (Yu et al., 2020).

Clinically, internal fixation of transplanted bone predominantly involves the use of reconstruction plates and titanium miniplates (Stanford-Moore and Murr, 2022). Reconstruction plates, characterized by robust fixation strength and broad adaptability, are commonly employed in complex defects, such as those crossing the midline or involving the condyle (Kreutzer et al., 2023). However, their large volume and stress-shielding effects may impact facial aesthetics and lead to bone resorption (Zhang et al., 2019). In contrast, titanium miniplates, with their small size, thin profile, and ease of application, have minimal impact on appearance, exhibit low stress-shielding effects, and are particularly suitable for simple defects and pediatric or adolescent patients to minimize interference with mandibular development (Graillon et al., 2023). Nevertheless, their fixation strength may be insufficient for multi-segmental repairs or stress-concentration areas (Graillon et al., 2022). However, existing studies have not yet systematically evaluated the influence of different fixation methods on vascularized iliac crest flaps and their clinical outcomes.

To address the question “Can titanium miniplates provide superior fixation for iliac crest flaps?” The study systematically compared the fixation stability, applicability, and complication profiles of titanium miniplates and reconstruction plates. This study aims to: (1) Identify defect types and segment numbers where titanium miniplates may outperform reconstruction plates, (2) Assess differences in complication rates between the two fixation methods, and (3) Optimize fixation method selection based on defect characteristics, thereby providing evidence-based guidance for clinicians to improve mandibular reconstruction outcomes.

## 2 Materials and methods

**FIGURE 1:**
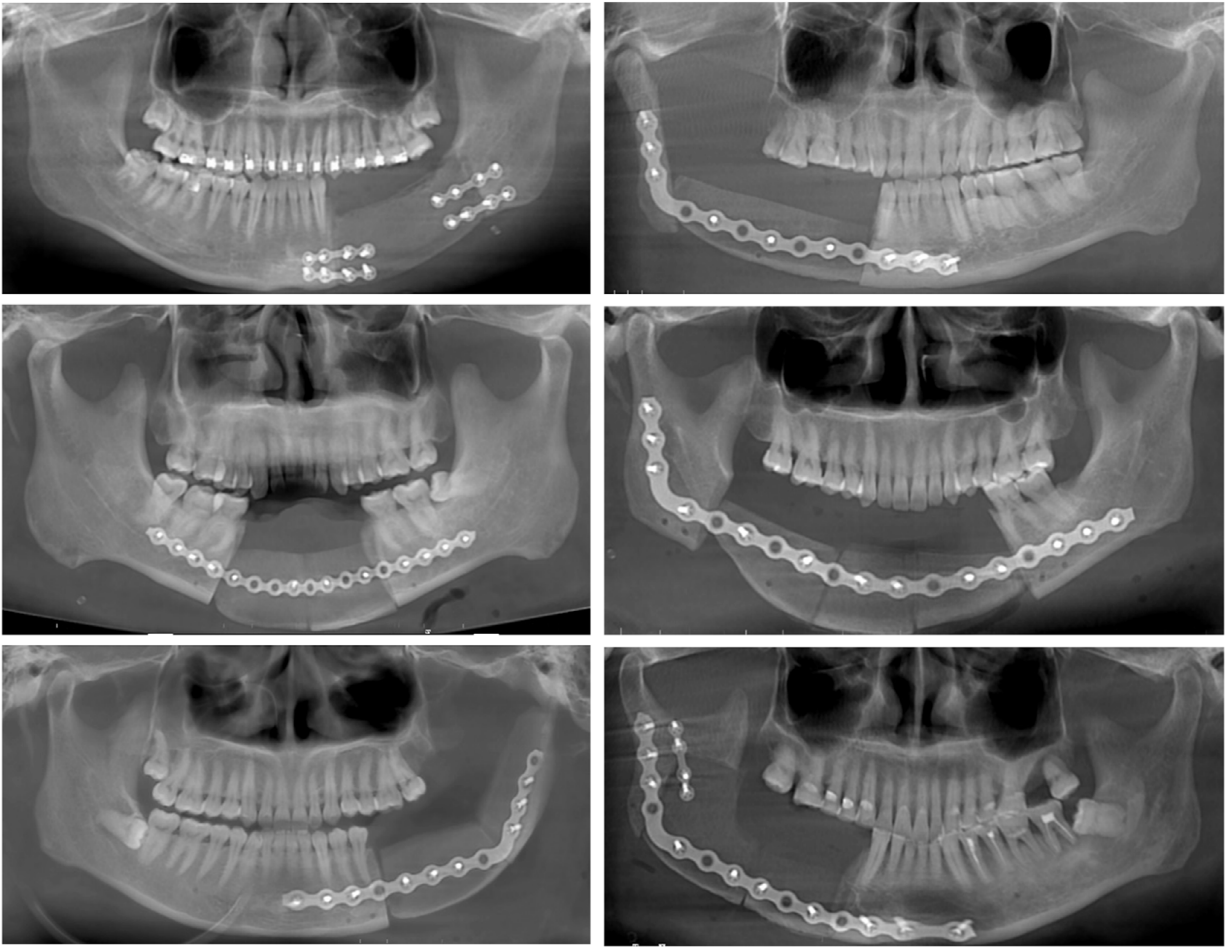
Demonstration of cases with different types of mandibular defects and number of grafted bone segments using two types of internal fixation: reconstruction plate and small titanium plate.

This study included 112 cases of mandibular defect reconstruction using vascularized iliac crest flaps performed at the Department of Oral and Maxillofacial-Head and Neck Oncology Surgery, School of Stomatology, Wuhan University, between January 2019 and December 2022 (Figure 1). Before surgery, all patients underwent high-resolution spiral CT scans of both the maxillofacial and iliac regions to obtain accurate anatomical data. The imaging data were processed using Mimics 19.0 software (Materialise, Belgium) to generate three-dimensional models of the donor and recipient sites. Surgeons collaborated with biomedical engineers to perform virtual osteotomies and simulate bone resection based on the lesion extent and reconstructive requirements. Customized surgical guides for mandibular resection, iliac bone harvesting, contouring, and graft positioning were then digitally designed. These guides were fabricated using 3D printing to assist in accurate intraoperative execution. In addition, titanium reconstruction plates were pre-bent manually according to the 3D-printed postoperative model, enabling precise transfer of the preoperative virtual plan to the actual surgery and ensuring accurate alignment of the bone segments.

The inclusion criteria of the study were: (1) complete electronic medical records, preoperative and postoperative imaging data, and pathological diagnoses; (2) postoperative follow-up of at least 1 year. Exclusion criteria were: (1) severe preoperative systemic diseases; (2) incomplete follow-up data. The study was approved by the hospital ethics committee (2023 B23) and registered at the Clinical Trial Registry Center on 18 July 2023 (ChiCTR2300073660), with written informed consent obtained from all participants for the use of their data in this research.

The mandibular defect types were classified according to Brown’s classification system, specifically as mandibular lateral defect (Type I), hemimandibulectomy (Type II), anterior mandibulectomy (Type III), and extensive anterior mandibulectomy (Type IV) (Brown et al., 2016). Postoperative quality of life was evaluated using the University of Washington Quality of Life Questionnaire (UW-QoL) (Hassan and Weymuller, 1993; Lin et al., 2025a; Lin et al., 2025b). Complications, including plate or screw exposure, loosening, or fracture, were identified through postoperative computed tomography (CT) follow-up and physical examinations. All statistical analyses were performed using SPSS Statistics software (version 26.0; IBM Corp., Armonk, NY, United States). Based on the results of normality testing, variables with a normal distribution were analyzed using one-way analysis of variance (ANOVA), whereas non-normally distributed variables were evaluated using the Kruskal–Wallis H test. Categorical variables were compared using the chi-square test or Fisher’s exact test. Data are presented as mean ± standard deviation or median (interquartile range), as applicable. A *p* value <0.05 was considered statistically significant.

## 3 Results

### 3.1 Patient characteristics

This study enrolled 112 patients who underwent single-stage reconstruction of segmental mandibular defects with vascularized iliac crest flaps, with iliac bone harvest lengths ranging from 4.49 to 13.2 cm. Four cases included skin islands or internal oblique muscles for soft tissue defect repair. The cohort comprised 47 males and 65 females, with ages ranging from 11 to 74 years. Pathological diagnoses primarily included ameloblastoma, odontogenic keratocyst, gingival carcinoma, and floor-of-mouth carcinoma (Table 1). The follow-up rates in the three groups were 85.7%, 78.6%, and 71.4%, respectively, and the follow-up duration showed no statistically significant difference (p = 0.620).

**TABLE 1:** Patient demographics.

Items	Reconstruction plate group (n = 77)	Mini plate group (n = 28)	Reconstruction + mini plate group (n = 7)	*P* value
Age (year)	44.74 ± 15.19	41.68 ± 13.79	49.14 ± 10.16	0.422^a^
Gender n (%)				0.896^b^
Male	31 (40)	13 (46)	3 (43)	
Female	46 (60)	15 (54)	4 (57)	
Pathological type				0.01^b^
Benign lesions n (%)	53 (69)	26 (93)	7 (100)	
Ameloblastoma	32	18	3	
OKC	8	4	2	
Others	13	4	2	
Malignant lesions n (%)	24 (31)	2 (7)	0	
OSCC	21	0	0	
Others	3	2	0	

^a^ One-way analysis of variance (ANOVA).

^b^ Fisher’s exact test.

### 3.2 Perioperative characteristics

As shown in Table 2, the operative time was the shortest in the group treated with mini plates alone, whereas the postoperative drainage duration did not differ significantly among the three groups (p = 0.958). Reconstruction plates (either alone or combined with mini plates) were more frequently applied in multi-segmental iliac crest flap reconstructions, while mini plates were predominantly used in single-segmental cases. The distribution of defect classifications showed slight variations among the groups. In the reconstruction plate group, four patients received skin paddles, but the use of skin islands did not reach statistical significance between groups.

**TABLE 2:** Perioperative patient characteristics.

Items	Reconstruction plate group (n = 77)	Mini plate group (n = 28)	Reconstruction + mini plate group (n = 7)	*P* value
Operation duration (day)	350.18 ± 85.35	253.07 ± 66.44	448.43 ± 57.27	<0.001^a^
Drainage duration (day)	6 (5.5∼8)	7 (6∼7)	6 (5∼7)	0.958^b^
Segmental status n (%)				0.003^c^
One-segment	47 (61)	23 (82)	1 (14)	
Two-segment	30 (39)	5 (18)	4 (57)	
Three-segment	0	0	2 (29)	
Defect length (mm)	86.1 (73.55∼94.8)	79.8 (59.8∼93.68)	86.9 (68.5∼102)	0.122^b^
Defect height (mm)	32.2 (29∼36.85)	31.95 (30.45∼35.55)	31.4 (28∼36.2)	0.873^b^
Defect classification n (%)				0.03^c^
Brown I(c)	56 (73)	13 (46)	2 (33)	
Brown II	13 (17)	12 (43)	3 (50)	
Brown III	8 (10)	3 (11)	1 (17)	
Skin paddle n (%)	4 (5.2)	0	0	0.670^c^

^a^ One-way analysis of variance (ANOVA).

^b^ Kruskal–Wallis H test.

^c^ Fisher’s exact test.

### 3.3 Quality of life assessment

The University of Washington Quality of Life Questionnaire (UW-QoL) was used to evaluate patients’ postoperative recovery (Table 3). Among the 12 assessed domains, only swallowing showed a statistically significant difference among the three groups, with the reconstruction plate group scoring significantly lower than the mini plate group (p = 0.009). For chewing, slight variations were observed among groups, but the difference did not reach statistical significance (p = 0.06). In other key domains such as pain, appearance, and speech, no significant differences were found between the groups. To exclude the potential impact of malignant tumors on QoL scores in patients reconstructed with reconstruction plates, we separately analyzed benign cases from both groups. The results were consistent with the overall QoL findings, as shown in Supplementary Table S1.

**TABLE 3:** UW-QoL assessment.

Questionnaire domains	Reconstruction plate group (n = 66)	Mini plate group (n = 22)	Reconstruction + mini plate group (n = 5)	*P* value
Pain	92.12 ± 9.85	93.6 ± 11.36	100	0.225^a^
Appearance	81.21 ± 12.09	76.36 ± 11.77	84 ± 8.94	0.2^a^
Activity	85.45 ± 17.38	88.18 ± 13.32	100	0.143^a^
Recreation	89.39 ± 16.54	93.63 ± 9.53	92 ± 10.95	0.505^a^
Swallowing	90.53 ± 13.7	98.86 ± 5.33	100	0.009^a^
Chewing	80.45 ± 18.84	84.55 ± 17.33	100	0.06^a^
Speech	93.18 ± 11.22	94.32 ± 10.72	95 ± 11.18	0.877^a^
Shoulder	95.45 ± 12.33	96.59 ± 8.78	100	0.659^a^
Taste	94.7 ± 13.53	96.59 ± 8.78	100	0.571^a^
Saliva	89.39 ± 17.57	96.59 ± 11.69	95 ± 11.18	0.176^a^
Mood	88.79 ± 16.13	93.63 ± 9.53	96 ± 8.94	0.274^a^
Anxiety	87.2 ± 15.91	89.77 ± 14.76	100	0.183^a^
Global quality of life score	81.52 ± 16.94	80 ± 17.46	92 ± 10.95	0.354^a^
Follow-up duration (mon)	17.97 ± 8.62	20.18 ± 10.4	18.2 ± 11.12	0.620^a^

^a^ One-way analysis of variance (ANOVA).

The follow-up rate in three groups were 85.7%, 78.6%, 71.4% respectively.

### 3.4 Complications and dental restoration

Fixation-related complications mainly included plate or screw exposure, loosening, and plate fracture, which were typically manifested by the development of fistulas with purulent discharge and confirmed through postoperative imaging examinations. The detailed incidence of these complications is summarized in Table 4. In addition, we investigated the prosthodontic rehabilitation status of patients in each group, and the overall rate of successful dental restoration was generally low across all groups.

**TABLE 4:** Complications and dental restoration status.

Item n (%)	Reconstruction plate group (n = 66)	Mini plate group (n = 22)	Reconstruction + mini plate group (n = 5)	P-value
Complication				
Plate/Screws exposure	4 (6)	2 (9)	0	0.742^a^
Plate/Screws loosening	2 (3)	1 (4.5)	0	1.000^a^
Plate fracture	0	0	0	-
Dental restoration				
In total	10 (15)	6 (27)	2 (40)	0.137^a^
Implant denture	8 (12)	4 (18)	2 (40)	-
Removable denture	2 (3)	2 (9)	0	-

The follow-up rate in three groups were 85.7%, 78.6%, 71.4% respectively.

^a^ Fisher’s exact test.

## 4 Discussion

The use of iliac crest flaps pedicled on the deep circumflex iliac vessels, combined with titanium plate fixation, has become a mainstream approach for mandibular defect reconstruction (Garajei et al., 2021). In clinical practice, long-span reconstruction plates, mini plates, or a combination of both are commonly employed (Wang et al., 2024). This study provides a large-scale retrospective analysis of patients treated with these three fixation strategies, aiming to offer practical insights into their clinical application and outcomes.

The choice of fixation method in mandibular reconstruction extends beyond a purely technical decision and reflects a balance between biomechanical stability, surgical accessibility, and anatomical restoration (Wang et al., 2024). In general, reconstruction plates remain indispensable for large or load-bearing defects, as their rigidity ensures structural integrity under functional stress. However, their bulkiness and potential stress-shielding effects necessitate cautious use in regions where aesthetic contour and bone vitality are critical (Urken et al., 1998; Robey et al., 2008; Knitschke et al., 2022). Mini plates, on the other hand, offer a less invasive and more flexible option in anatomically confined spaces, allowing precise adaptation with minimal interference to surrounding tissues (Robey et al., 2008). The selective or combined use of both fixation systems underscores the importance of individualized planning—tailoring fixation to the defect’s geometry, functional requirements, and the surgeon’s intraoperative assessment. Rather than suggesting superiority of one method over another, our findings highlight that appropriate fixation selection, guided by defect characteristics and reconstructive principles, may be more decisive for long-term outcomes than the fixation choice.

From clinical perspective, although operative time varied among fixation methods, this difference mainly reflects surgical complexity rather than technical superiority, as mini plates were generally applied to less extensive, single-segmental reconstructions. The comparable drainage duration and similar rates of major complications—such as plate exposure, loosening, or fracture—suggest that both fixation systems offer acceptable safety profiles when appropriately selected. Postoperative UW-QoL assessment showed that although there was no significant difference in overall quality of life among the three groups, variations were observed in specific domains such as swallowing. The difference in swallowing scores between fixation methods may be attributed to variations in defect morphology, surgical exposure, and perioperative soft-tissue management. Mini plates are typically used in more localized reconstructions, causing less disruption to the oropharyngeal musculature and facilitating earlier restoration of swallowing coordination. In contrast, reconstruction plates are generally applied in anatomically more complex reconstructions, where wider surgical dissection and postoperative stiffness may transiently affect swallowing function. These factors collectively may account for the observed differences rather than the fixation hardware itself.

The rapid advancement of computer-assisted surgical technology has greatly enhanced the precision and predictability of vascularized bone flap transplantation (van Baar et al., 2021; Li et al., 2020; Shen et al., 2012). By acquiring high-resolution spiral CT scans of both the donor and recipient sites, patient-specific three-dimensional models can be generated, allowing for personalized virtual surgical planning through close collaboration between surgeons and biomedical engineers. The use of customized surgical guides—including cutting guides for mandibular resection, harvesting guides for iliac bone, and positioning guides for flap placement—has significantly improved intraoperative accuracy and reduced technical variability. Such digital integration facilitates precise bone alignment and tension-free fixation, thereby promoting reliable bone union. Mini plates are considered more favorable for promoting bone healing (Robey et al., 2008; Steffen et al., 2023). A study has shown that, in the short-term postoperative period, mini plates can achieve better bone healing compared with reconstruction plates; however, in the long term, the differences between the two fixation methods are not significant (Wang et al., 2024). In our series, no cases of apparent osseous non-union were observed during follow-up, reflecting the practical benefit of meticulous preoperative design rather than fixation technologies. These findings highlight how digital technology serves as a powerful adjunct in optimizing flap design and fixation accuracy, contributing to safer and more reproducible reconstructive outcomes.

This study has several limitations. As a retrospective analysis, it was not possible to completely eliminate the influence of surgeon preference in the selection of fixation methods. Nevertheless, as discussed above, fixation choice in our center is largely determined by the defect’s extent, anatomical location, and functional demands rather than by individual bias. In addition, due to the limited number of malignant cases within the mini plate group, subgroup comparisons based solely on pathology were of limited statistical value. To minimize this effect, we performed a separate analysis including only benign lesions, and the results remained consistent with the overall QoL findings (see Supplementary Table S1). Furthermore, the “Reconstruction + Mini Plate” subgroup had a small sample size for QoL and complication analysis, which may lead to insufficient statistical power. Despite our best efforts to conduct comprehensive postoperative follow-up, the total sample size remains limited, and long-term outcomes require validation in larger, prospective, multi-center studies. Future research integrating objective functional assessments and longitudinal quality-of-life data will be essential to further substantiate and refine these findings.

## 5 Conclusion

In summary, this study provides a descriptive overview of clinical outcomes associated with different fixation strategies used in mandibular reconstruction with vascularized iliac crest flaps. In our cohort, mini plates were typically selected for single-segment defects, whereas reconstruction plates were used for multi-segmental defects requiring enhanced mechanical stability. These choices reflected standard surgical practice rather than experimental allocation. Both fixation approaches yielded satisfactory postoperative outcomes, and no significant differences were observed in long-term overall quality of life. However, a statistically significant difference was noted in the “Swallowing” domain, where patients treated with mini plates reported better postoperative swallowing function, which may be related to differences in defect morphology and surgical exposure rather than the fixation device itself. Therefore, the present findings should be interpreted as reflecting real-world applications of fixation methods tailored to defect characteristics, rather than as evidence of the superiority of one technique over the other.

## Data Availability

The original contributions presented in the study are included in the article/Supplementary Material, further inquiries can be directed to the corresponding authors.
