# Supplementary material for: Can titanium miniplates provide superior fixation compared to reconstruction plates in mandibular repair with iliac crest flaps? A retrospective study
Source: Front Bioeng Biotechnol. 2025 Dec 2;13:1688458. doi: 10.3389/fbioe.2025.1688458 (PMC12705601; doi:10.3389/fbioe.2025.1688458)
Supplement: Supplementary file 1 [file Table1.docx]

**Can Titanium Miniplates Provide Superior Fixation Compared to Reconstruction Plates in Mandibular Repair with Iliac Crest Flaps?**

**: A Retrospective Study**

Wen-qing Lin^3,4#^, Da Liu^3,4#^, Renbin Zhou^1^, Hao Lin^1^, Bang Zeng^1^, Jun Jia^1,2^, Yifang Zhao^1,2^, Bing Liu^1,2*^, Tianfu Wu^1,2*^

^1^State Key Laboratory of Oral & Maxillofacial Reconstruction and Regeneration, Key Laboratory of Oral Biomedicine Ministry of Education, Hubei Key Laboratory of Stomatology, School & Hospital of Stomatology, Wuhan University, 237 Luoyu Road, Hongshan District, Wuhan 430079, China.

^2^Department of Oral and Maxillofacial Head Neck Surgery, School & Hospital of Stomatology, Wuhan University, 237 Luoyu Road, Hongshan District, Wuhan 430079, China.

^3^Department of Oral and Maxillofacial Surgery, Stomatological Hospital of Xiamen Medical College.

^4^Xia-men Key Laboratory of Stomatological Disease Diagnosis and Treatment. Xiamen 361008, Fujian Province, China

#: Wen-qing Lin and Da Liu contributed equally to this research article.

**Corresponding Author:**

Bing Liu

Email: liubing9909@whu.edu.cn

Tianfu Wu

Email: wutianfu@whu.edu.cn

**Keywords:** Titanium miniplate, Reconstruction plate, Mandibular reconstruction, Vascularized iliac crest flap, Complication

**Supplementary Table 1. UW-QoL assessment of benign tumor patients**

| **Questionnaire domains** | **Reconstruction Plate Group (n=47)** | **Mini Plate Group (n=20)** | | **Reconstruction + Mini Plate Group (n=5)** | ***P* value** |
| --- | --- | --- | --- | --- | --- |
| Pain | 91.4±9 10 | 93±11.74 | 100 | | 0.210 |
| Appearance | 82.13±11.22 | 77±11.74 | 84±8.94 | | 0.196 |
| Activity | 83.4±18.33 | 88±13.61 | 100 | | 0.09 |
| Recreation | 87.66±18.44 | 93±9.79 | 92±10.95 | | 0.437 |
| Swallowing | 89.36±14.5 | 98.75±5.59 | 100 | | 0.009 |
| Chewing | 81.21±18.44 | 83±17.44 | 100 | | 0.084 |
| Speech | 93.61±11.01 | 93.75±11.1 | 95±11.18 | | 0.965 |
| Shoulder | 95.21±12.38 | 97.5±7.69 | 100 | | 0.530 |
| Taste | 93.61±15.17 | 96.3±9.16 | 100 | | 0.506 |
| Saliva | 91.49±15 | 96.25±12.23 | 95±11.18 | | 0.431 |
| Mood | 86.38±17.75 | 93±9.79 | 96±8.94 | | 0.167 |
| Anxiety | 85.21±16.97 | 88.75±15.12 | 100 | | 0.135 |
| Global quality of life score | 79.57±17.44 | 78±17.04 | 92±10.95 | | 0.255 |
| Follow-up duration (mon) | 18.34±8.18 | 20.4±10.67 | 18.2±11.12 | | 0.690 |

a. one-way analysis of variance (ANOVA);
